# Ethnozoological study of animals based medicine used by traditional healers and indigenous inhabitants in the adjoining areas of Gibbon Wildlife Sanctuary, Assam, India

**DOI:** 10.1186/s13002-017-0167-6

**Published:** 2017-06-30

**Authors:** Manash Pratim Borah, Surya Bali Prasad

**Affiliations:** Cell and Tumor Biology Laboratory, Department of Zoology, School of Life Sciences, North-Eastern Hill University, Umshing, Shillong, 793022 India

**Keywords:** Ethnozoology, Zootherapy, Traditional healers, Fidelity level

## Abstract

**Background:**

India has an immense faunal, floral, as well as cultural diversity with many ethnic communities who are primarily dependent on the traditional medicinal system for their primary health care. Documentation and evaluation of this indigenous remedial knowledge may be helpful to establish new drugs for human health. The present study is intended to look into different zootherapeutic medicinal uses in the traditional health care system among the native inhabitants adjacent to the Gibbon Wildlife Sanctuary, Assam, India.

**Methods:**

Field survey was carried out from March 2015 to August 2015 by personal interviews through semi-structured questionnaires. In some cases where participants were uncomfortable with the questionnaires, informal interviews and open group discussions were conducted with a total of 62 indigenous respondents (43 male and 19 female) who provided the information regarding various medicinal uses of animals and their products (local name of animal, mode of preparation, application etc).

**Results:**

The study recorded a total of 44 different species, 44 genera and 36 families of animals which are used for the treatment of 40 different ailments. Insects occupied the highest uses (30.9%), followed by mammals (23.8%), fishes (16.7%), reptiles (11.9%), amphibians (7.1%), annelids (4.8%) and gastropods (4.8%). Further, some zootherapeutic animals i.e. cockroach (*Periplaneta americana*), praying mantis (*Mantis religiosa*) and earthworms (*Metaphire houletti*, *Pheretima posthum*) are used for the treatment of asthma, otorrhoea and cancer respectively.

**Conclusion:**

The findings suggest that the traditional zootherapeutic remedial measures followed by the native people adjacent to Gibbon Wildlife Sanctuary plays an important role in their primary health care. The documentation of this indigenous knowledge on animal based medicines should be very helpful in the formulation of strategies for sustainable management and conservation of bio-resources as well as providing potential for the novel drugs discovery.

## Background

Bioresources involving both plants and animals have been used in the indigenous healing practices by different cultures since ancient time [1, 2]. In modern society also traditional medicinal knowledge constitutes an important alternative in health care system. About 70–80% of the world rural population depends on traditional medicine for its primary health care [3]. The percentage of the population using traditional medicines for primary health care is more (60–90%) in developing countries than that in developed countries (23–80%) [4]. Around 60% of commercially available drugs are based on bioactive compounds extracted from natural resources traditionally used by various indigenous cultures around the globe [5]. Although plants and plant derivatives have been used as a major constituent of traditional medicine, the identification of animal resources for medicinal cure is also important in human health care [1, 6].

Zootherapy is defined as healing of human ailments by using medicines prepared from different animals and/or animal derived byproducts [7]. Zootherapy constitutes a significant substitute among many other known therapies practiced worldwide [1]. In Latin America, 584 animals distributed in 13 taxonomic categories were recorded with traditional therapeutic medicinal value [8], while 283 animal species were reported to be used for the treatment of various ailments in Brazil [9]. In Bahia, the Northeast State of Brazil, over 180 animal species were recorded for the treatment in traditional health care practices [10]. The rural community in the semi-arid region of Northeastern Brazil were reported to use 51 animal species to treat different ailments [11]. Toba (qom) communities of Argentine Gran Chaco region have been documented to use 72 animal species belonging to 52 families as a part of animal pharmacopeia [12]. In Traditional Chinese medicine, more than 1500 animal species have been reported to be of some medicinal importance [13]. A review on the global traditional use of primates reported the use of 110 species of primates belonging to 41 genera and 11 families in traditional folk medicine and in magic-religious rituals [14]. Lev and Amar (2000) documented the use of 20 animal species as traditional drugs in Israel [15]. Alves and Rosa (2007) recorded 138 animal species being used in traditional medicine to treat 100 illnesses by the fishing communities of the North and Northeastern regions of Brazil [16]. An overview of the global traditional uses of reptiles revealed that at least 165 reptile species belonging to 104 genera and 30 families are used in traditional folk medicine around the world [17].

India has a great faunal diversity accounting about 10% of the reported biological species on the planet and ranks first place in terms of insects (54,600), followed by fishes (2546), aves (1232), reptiles (456), mammals (390) and amphibians (209) [18, 19]. Various zootherapeutic traditional medicines have been reported and documented in great historical books like Ayurveda and Charak Samhita in India. About 15–20% of the Ayurvedic medicines are based on animal derived substances [20]. Different tribes and ethnic communities inhabiting in different parts of India have a rich knowledge about animals and their medicinal value for their primary health care needs [21]. Therefore, it is utmost important to record the conventional indigenous knowledge of different ethnic communities as many rural communities are loosing their socioeconomic and cultural characteristics [22].

North-eastern region of India is inhabited by various ethnic groups and tribes with wide cultural diversity [23]. As per 2011 census, the North-eastern region is inhabited by a total of 427 tribal groups which have their own traditional cultural identity. There are only a few reports available from the region about the use of animals in traditional medicine. The traditional methods of treating various ailments using 81 species of edible and therapeutic insects and 36 vertebrate species by the Nyishi and Galo tribes of Arunachal Pradesh were reported [24, 25]. Twenty-six animal species were reported for the treatment of different diseases like asthma, tuberculosis, rheumatic pain, paralysis, etc. by different Naga tribes of Nagaland [26]. Recently, indigenous Khasi tribes of East Khasi hills district, Meghalaya were reported to use 13 animals against asthma, anemia, diarrhea, cough, fever etc. in their traditional zootherapeutic practices [27].

Among the eight Northeastern States of India, Assam is the second largest State having rich, unique ethnic and cultural diversity, richness in forest resources and wildlife sanctuaries. Many reports on the plant based traditional medicine used by the people of Assam have been documented [28]. However, only a few reports are available on the study of zootherapeutic remedial uses. Thirty-four different animal species have been recorded for the treatment of 34 different ailments among Biate tribes in the Dima Hasao district of Assam [29]. A total of 26 ethnomedicinal animals and animal products were accounted for the treatment of different diseases like jaundice, asthma, pneumonia, etc. among the indigenous inhabitants in adjoining areas of Pobitora Wildlife Sanctuary, Assam, India [30]. Among Karbi community, a total of 48 different animals were reported to be used for different therapeutic purposes against various diseases like piles, cancer, tuberculosis, eczema etc. [31].

The knowledge on the use of different animals in traditional medicine by different ethnic communities is generally passed orally from one generation to another generation and this knowledge is sometimes lost with the death of the elderly knowledgeable person. Nowadays, Indian traditional knowledge system is fast eroding due to urbanization. So, it is vital to study and document the ethnobiological information regarding the therapeutic use of different animals in traditional medicine among different ethnic communities before the traditional cultures are completely lost [32].

Many studies have been undertaken on Gibbon Wildlife Sanctuary related with the Hollock Gibbon conservation, faunal diversity and medicinal plants [33–35], however, there is no report available about its ethnozoological value utilized by the people inhabiting adjacent to this Sanctuary. Thus, the present study involving the documentation of traditional zootherapeutic medicinal remedies used by indigenous people inhabiting in the adjoining areas of Gibbon Wildlife Sanctuary was undertaken which may provide information in making strategies for sustainable utilization of natural resources and biodiversity and also protect traditional knowledge for future generation.

## Methods

### Study area

**Fig. 1:**
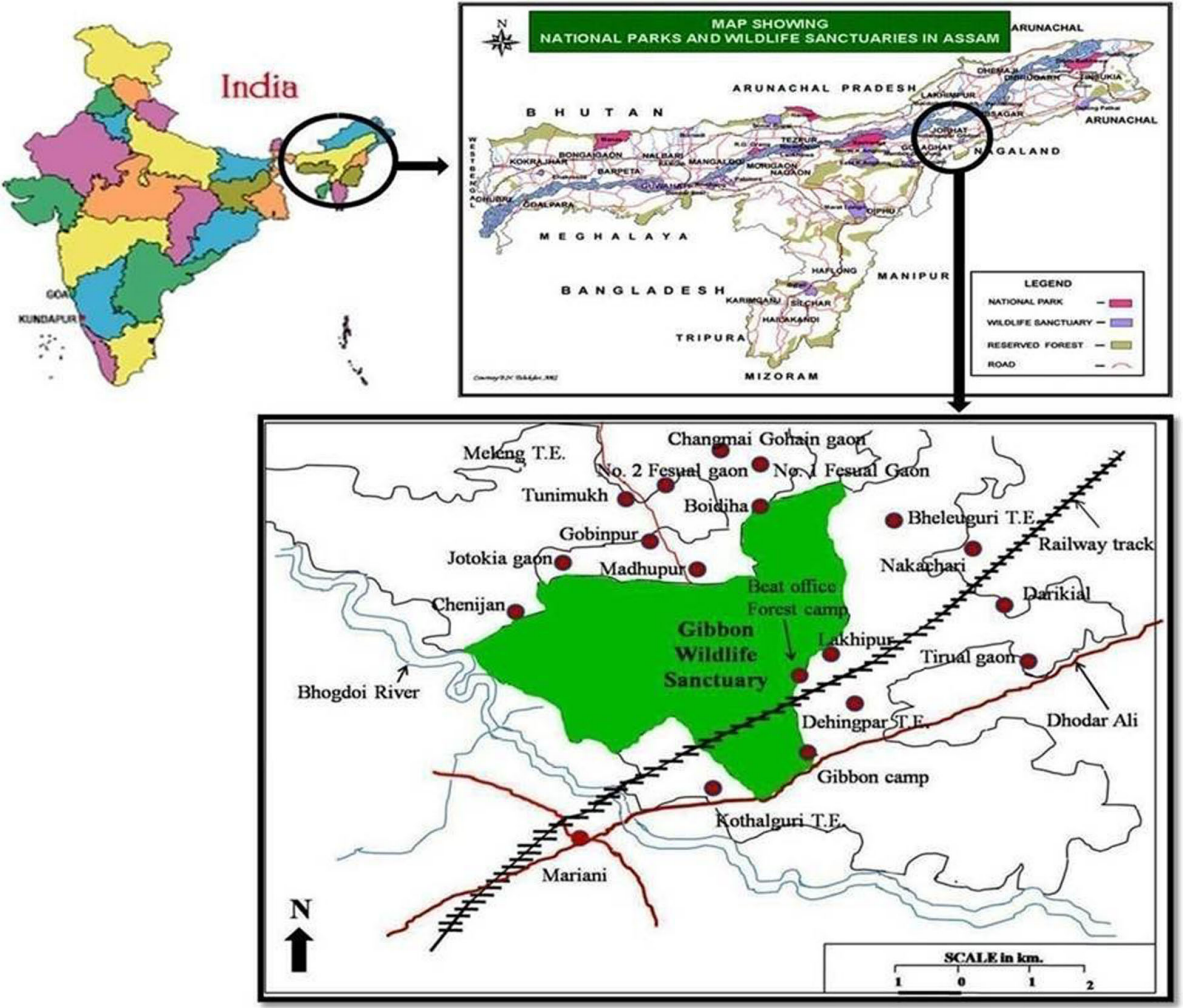
Map showing the sites of field survey ( marks)

Gibbon Wildlife Sanctuary is the only sanctuary named after non-human primate the Hoolock Gibbon (*Hylobates hoolock*). It is located in the close proximity of the Naga Hills in the Jorhat district of Assam, India and covers an area of about 20.98 sq. km. extended between latitude 26°40′ N to 26°45′ N and 94°20′ E to 94°25′ E longitude (Fig. 1). The sanctuary is topographically characterized by an almost level land with an average contour height of 90 m from mean sea level with an average annual rainfall of 249 cm [36]. The sanctuary is famous for harbouring seven species of primates namely Western Hoolock Gibbon (*Hoolock hoolock*), Stump-tailed Macaque (*Macaca arctoides*), Eastern Assamese macaque (*Macaca assamensis*), Northern Pig-tailed macaque (*Macaca lenina*), Bengal Slow Loris (*Nycticebus bengalensis*), Indian Rhesus macaque (*Macaca mulatta*) and Capped Langur (*Trachypithecus pileatus durga*) [36]. The other main animals present are Indian elephant (*Elephas maximus*), sambar (*Cervus unicolor*), wild boar (*Sus scrofa*), jungle cats (*Felis chaus*), leopards (*Panthera pardus*), four types of squirrel, etc. The Sanctuary is harboured by other types of mammals, 232 species of birds and several types of snakes [34, 36].

### Socio-cultural diversity around gibbon wildlife sanctuary

Gibbon Wildlife Sanctuary exhibits a great ethnic cultural diversity surrounded mainly by human settlement and tea gardens. The major ethnic communities inhabited adjacent to wildlife sanctuary are Ahom, Chutiya, Koch-Rajbonshi, Kalita and tea tribes (Adivasi). Ahom, Chutiya and Koch-Rajbonshi people belong to Mongoloid groups. However, Kalita community of Assam commonly claimed themselves to belong to the Kshatriya caste and they considered as pure Aryans and it was thought that they were the first to introduce the Aryan culture in Assam [37, 38]. The tea tribes of Assam (Adivasi) are the people who were brought to the State in the British era as workers in tea gardens by colonial tea planters. The community consists of people belonging to the indigenous tribal community like Munda, Santhal. Bhumiz etc. Although all these communities are from different origin, nevertheless they are patriarchal by nature and belong to Assamese and use Assamese scripts [39].

The present study was conducted in the villages surrounding the Gibbon Wildlife Sanctuary and the information was collected mainly from the people of Ahom, Chutiya, Koch-Rajbongsi and Kalita communities.

### Data collection

Field surveys were conducted in villages surrounding Gibbon Wildlife Sanctuary from March to August 2015. The ethnomedicinal data about the use of animals and their products were collected using the participatory rural appraisal (PRA) method, where the informants also sometimes become investigator themselves, involves an interview, informal meetings, open and group discussions and with semi-structured questionnaires [40–42].

During survey, details were asked in semi questionnaire form on the ethnozoological information, including local name of animal, part used, ailments, method of preparation, mode of administration, dosage, duration of treatment etc. concerning each of the traditional medicine [41–44]. The age of respondents varied from 30 to 80 years. A total of 62 individuals were interviewed. The respondents/informants were selected mainly on the basis of their experience, recognition as an expert knowledgeable persons, traditional healers concerning traditional medicine. Moreover, the detailed ingredients of medicine whether they use only animal parts or mixed with other ingredients like plant material were also noted. The scientific name and species name of animals were identified using relevant standard literature [45, 46] and also in association with Zoological Survey of India (ZSI), Shillong.

### Data analysis

#### Relative frequency of citation

Relative frequency of citation (RFC) index shows the local importance of each species. The RFC value was calculated using the formula RFC = FC/N; where FC is the number of informants mentioning about the use of the species and N is the number of informants participating in the survey [47]. This RFC index varies from 0 to 1. When RFC index is 0, it means that no one refers to the animal as useful and when RFC index is 1, it indicates that all informants in the survey refer to the animal as useful [48].

#### Fidelity level

For the data analysis, fidelity level (FL) was calculated to determine the most commonly used animal species in the treatment of a particular disease category by the informants of the study area. Fidelity level is useful for identifying the resident’s most preferred species in use for treating certain ailments. The FL was calculated [49] by using the formula as follows:

$$ \mathrm{FL}\left(\%\right)= N\mathrm{p}\times 100/\mathrm{N} $$

Where *N*p is the number of informants that claim a use of a specific animal species to treat a particular ailment and *N* is the total number of the informants who utilized the animals as a medicine to treat any given disease.

## Results and discussion

### Demographic details of informants

The inhabitants in villages surrounding the Sanctuary have a strong belief and knowledge regarding the source and use of traditional medicine. They use different plants, animals and animal byproducts for curing different ailments in their own indigenous ways. The knowledge regarding traditional medicine is usually confined to the local medicinal practitioners popularly known as Kabiraj, Bez and Bejini. Demographic information of the respondents was collected through face to face interaction. During the survey, respondents comprised an uneven distribution of the male-female ratio, where 69.4% respondents were male and only 30.6% were female. The high male-female ratio may indicate the dominancy of the participation of male medicinal practitioners over female medicinal practitioners. The same trend was also reported in other studies [30, 31, 50]. The respondents belonged to mainly 4 ethnic groups i.e. Ahom, Chutiya, Koch-Rajbangsi and Kalita communities with the highest number of respondents from Ahom community (41.9%) (Table 1).

**Table 1:** Demographic profile of the informants included in survey (*N* = 62)

Demographic features	Number of people	Percentage (%)
Gender		
Male	43	69.4
Female	19	30.6
Education		
Primary education	9	14.5
Secondary education	36	58.1
Graduate	17	27.4
Extra qualification	0	
Religion		
Hindu	62	100
Muslim	0	
Christian	0	
Ethnicity		
Ahom community	26	41.9
Chutiya community	11	17.7
Koch- Rajbangsi community	9	14.5
Kalita community	16	25.8
Age of traditional healer		
Between 31 and 40 years	4	6.5
Between 41 and 50 years	9	14.5
Between 51 and 60 years	20	32.3
Between 61 and 70 years	26	41.9
Above 70 years	3	4.8

The age of the respondents varied from 31 to 80 years. The percentage of local medicinal practitioner with age lower than fifty was found to be very less with only 21% as compared to 79% of the aged group of society above 50 years (Table 1). The demographic table of the respondents showed that the aged groups of the society were more knowledgeable about traditional medicinal uses than that of younger generation. This trend was very similar to the observation in other region of Assam made by Verma et al. [31] and may also indicate that the aged people were more experienced in the zootherapeutical practices which were passed to them by their elders. The reason of less traditional medicinal knowledge among the younger generation could be due to urbanization and assimilation of alien culture.

Most of the respondents had secondary level education while some of them were up to graduation level (Table 1). Only 12 respondents (19.4%) were formally employed in government sector mainly as school teachers while others were mostly farmers, workers and local traditional healers. Most of the informants practiced this traditional therapy as a part time job to serve the society. However, some are renowned well known herbalist/healers who practice this traditional medicinal knowledge in large scale as their profession.

### Ethnozoological analysis

**Fig. 2:**
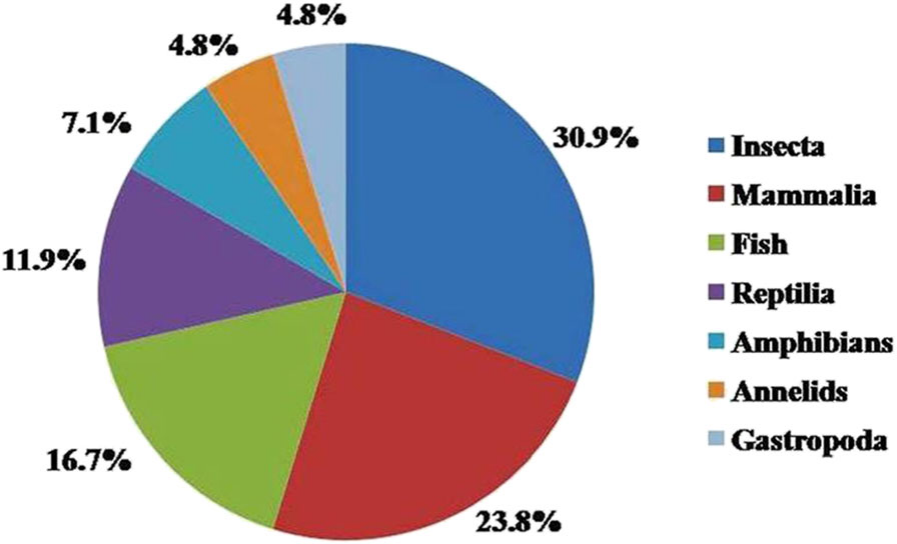
Percentage of animal categories being used in zootherapeutic practices among the traditional healers in the adjoining areas of Gibbon Wildlife Sanctuary

The study recorded a total of 36 families, 44 genera, and 44 species of animals which were used to treat 40 different disease conditions. Table 2 summarizes the English name, scientific name, local name, the parts or byproduct of the species used to treat the disease(s) or ailment(s). These 44 animal species belonged to both verterates (25 species) and invertebrates (19 species). These animal species belong to 42 taxonomic groups among which insects occupied a highest number of animals (30.9%), followed by mammals (23.8%), fish (16.7%), reptiles (11.9%), amphibians (7.1%), annelids (4.8%) and gastropods (4.8%) (Fig. 2). Insects may have been used mostly because of the easy availability in the study area, as was reported from Arunachal Pradesh, India [24]. The second highest zootherapeutic animals to be used are mammals as some of them are domesticated animals. However, in some reports mammals and reptiles are among the main group of animals used in folk medicine [17, 51, 52]. The use of mammals in traditional medicine has also been reported from other parts of India [30, 31, 53]. This finding demonstrates the importance of local faunal diversity in furnishing folk medicine as suggested by Alves and Rosa [16] who observed that faunal composition, accessibility and availability directly influence the type of zootherapeutic resources used in any given region.

**Table 2:** Medicinal uses of animals and animal parts for traditional therapeutic purposes by the people inhabiting adjacent area of Gibbon Wildlife Sanctuary

No.	Animal group	*Local name	English name	Scientific name	Family	Part used	Application	Medicinal use	Prescription	RFC	FL (%)
1	Insect	Kumoti	Mole cricket	*Scapteriscus borellii* (Giglio-Tos, 1894) (IV)	Gryllotalapidae	Alimentary canal	Oral	Intestinal worm (thread worm)	Raw alimentary canal part is consumed	0.32	47.4
2	Insect	Junaki paruwa	Fire Flies	*Lampyridae spp.* (Latreille, 1817) (IV)	Lampyridae	Whole body	Oral	Cancer	4/5 raw fire flies prescribed to eat daily	0.26	68.6
3	Insect	Poitasura	Cockroach	*Periplaneta Americana* (Linnaeus, 1758) (IV)	Blattidae	Whole body	Oral	Asthma	Wings are removed and washed, then boiled with water and prescribed to consume	0.65	92.3
4	Insect	Mou makhi	Honey bee	*Apis cerna indica* (Fabricius, 1793) (IV)	Apidae	Whole body	Oral	Cancer	Whole honey bee is ground in water and prescribed to eat.	0.19	38.9
5	Insect	Kodu	Hornet	*Vespa affinis* (Linnaeus, 1764) (IV)	Vespidae	Whole body	Oral	Cancer	It is ground and mixed with water and prescribed to drink	0.08	18.5
6	Insect	Kola paruwa	Weaver ant	*Myrmicaria brunnea* (Saunders, 1842) (IV)	Formicidae	Whole body	Oral	Body pain	Prescribed to eat raw	0.13	46.2
7	Insect	Mojali poruwa	Slender ant	*Tetraponera rufonigera* (Jordan, 1851) (IV)	Formicidae	Whole body	Oral	Body ache	Prescribed to eat raw	0.05	25.0
8	Insect	Amoli poruwa	Green tree ant	*Oecphylla smaragdina* (Fabricius, 1775) (IV)	Formicidae	Whole body	Oral	Sinus	Prescribed to eat raw	0.11	65.0
								Cancer	Prescribed to eat raw		44.4
								Epistapix (bleeding from nose)	Ant is fried and prescribed to eat		66.7
9	Insect	Gandhi puk	Gundhi kira (Rice bug)	*Leptocorisa varicornis* (Fabricius, 1787) (IV)	Alydidae	Whole body	Oral	Fever	Whole insect is boiled and prescribed to consume	0.21	42.9
10	Insect	Muga palu	Muga silk worm	*Antheraea assamensis* (Helfer, 1837) (IV)	Saturnidae	Whole body	Oral	-	Eat raw because of its high protein content	0.31	100
11	Insect	Uisiringa	House cricket,	*Acheta domestica* (Linnaeus, 1758) (IV)	Gryllidae	Whole body	Oral	Pain,	Fried and prescribed to eat	0.06	63.2
								For better eye sight			68.7
								improve pancreas			40.0
12	Insect	Poda paruwa	Bombardier beetle	*Pheropsophus spp.*(Solier, 1833) (IV)	Carabidae	Whole body	Oral	Alcoholic habit	Raw beetle is consumed	0.02	6.9
13	Insect	Gagini foring	Praying mantis	*Mantis religiosa* (Linnaeus, 1758) (IV)	Mantidae	Cocoon with larva	Topical	Otorrhoea (Wound in ear)	Cocoon with larvae is burned and ash is mixed with coconut oil and prescribed to apply in wounded area directly with feather	0.27	80.0
						Whole insect	Oral	Pneumonia	Insects are ground into powder and mixed with milk and prescribed to drink		62.5
14	Annelida	Bonda kesu	Earthworm	*Metaphire houletti* (Perrier, 1875) (IV) *Pheretima posthum* (Vaillant, 1868) (IV)	Megascolecidae	Whole body	Oral	Vocal chord infection	Earthworm is washed properly and boiled with water, salt is added and prescribed to consume.	0.68	80.0
						head	Oral	piles	Head portion of earthworm is inserted in goroi fish after removing alimentary canal part and fried and prescribed to eat		78.9
						head	Topical	piles	Head portion is burned and grind to powder, then mixed with coconut oil and 4–5 leaves of Jetulipoka (*Rubus rugosus* Sm.) and a paste is made. Then the paste is prescribed to use externally in piles		83.3
						Whole body	Oral	Cancer	Raw earthworm are squished and prescribe to consume		85.7
15	Annelida	kesu	Earthworm	*Perionyx spp.* (Perrier, 1872) (IV)	Megascolecidae	Whole body	Oral	Pneumonia	3 earthworms are ground, then mixed with salt and prescribed to eat for 2 days twice a day	0.16	95.2
16	Gastropod	kunjelekuwa	Snail	*Cryptozona bistrialis* (Beck, 1837) (IV)	Ariophantidae	Whole body	Oral	Impotence (to increase sexual power)	Boiled and prescribed to eat	0.03	26.7
17	Gastropod	Shamuk	Freshwater snail	*Pila spp.*(Roding, 1798) (IV)	Ampullaridae	Whole body	Oral	For better eye sight	Raw snail is eaten	0.58	84.0
						Soft watery portion	Topical	Pain	Applied externally for massage		63.2
								bone fracture			46.7
18	Amphibian	Pat beng	Common tree frog	*Polypedates leucomystax* (Gravenhorst, 1829) (V)	Rhacophoridae	Meat	Oral	Asthma	Meat is boiled with spices like Clove, Cinnamon, black pepper and prescribed to eat	0.05	17.2
19	Amphibian	Suk vekuli	Common toad	*Bufo spp.* (Linnaeus, 1758) (V)	Bufonidae	heart with blood	Oral	Bronch pneumonia (bolianerengia)	Fresh blood and heart mixed with clove, cardamom, pepper a paste is made and prescribed to consume	0.47	54.5
20	Amphibian	Pani vekuli	Frog	*Ranna spp.* (Linnaeus, 1758) (V)	Ranidae	Meat	Oral	Asthma	Meat is cooked and prescribed to eat	0.10	42.9
21	Fish	Rou mas	Rohu	*Labeo rohita* (Hamilton, 1822) (V)	Cyprinidae	Gall bladder (Bile)	Oral	Gastric	Ground with water and prescribed to eat whole thing	0.15	57.9
								Gastric ulcer,			41.7
								Intestinal cancer			57.1
22	Fish	Magur mas	Magur	*Clarias batrachus* (Linnaeus, 1758) (V)	Claridae	Whole body	Oral	Body ache	Cooked with spices like black pepper and prescribed to eat	0.59	66.7
								wound healing			52.6
23	Fish	Singhi mas	Stinging cat	*Heteropneustes fossilis* (Bloch, 1794) (V)	Heteropneustidae	Whole body	Oral	Pain	Cooked with spices like black pepper and prescribed to eat	0.47	63.2
								wound healing			44.0
24	Fish	Cuchia	Eel	*Amphipnous cuchia* (Hamilton, 1822) (V)	Synbranchidae	Whole body and blood	Oral	Aneamia	Raw blood is prescribed to drink and cooked meat is prescribed to eat	0.66	90.9
25	Fish	Chengeli mas	Assamese snake head	*Channa stewartii* (Playfair, 1867) (V)	Channidae	Whole body	Oral	Diabetes	Boiled in water and consumed	0.08	63.0
								Pain			53.9
								High pressure			48.0
26	Fish	Moa mas	Mole/Indian carplet	*Amblypharyngodon mola* (Hamilton, 1822) (V)	Cyprinidae	Whole fish	Oral	Pox	Cooked fish is prescribed to eat	0.26	75.0
								Pain			57.1
								Asthma			66.7
27	Fish	Kurkuri mas	Devil fish	*Chaca chaca* (Hamilton, 1822) (V)	Chacidae	Meat	Oral	Asthma	Dry fish is grind and prescribed to drink with water	0.04	81.2
28	Reptile	Musuwa shap	Indian rat snake	*Ptyas mucosa* (Linnaeus, 1758) (V)	Colubridae	Meat	Oral	Pain or body ache	Cooked meat is prescribed to eat	0.06	35.1
29	Reptile	Tezpia	Common Indian skink	*Eutropis carinata* (Schneider, 1801) (V)	Scincidae	Meat	Oral	Snake bite and pain	It is boiled in water and after boiling water portion is prescribed to drink	0.03	21.1
30	Reptile	gui	Bengal monitor	*Varanus bengalensis* (Daudin, 1802) (V)	Varanidae	Meat	Oral	Skin disease (ring worm)	Boiled meat is prescribed to eat.	0.29	70.0
31	Reptile	jethi	Indian wall lizard	*Hemidactylus flaviviridis* (Ruppell, 1835) (V)	Gekkonidae	Meat	Oral	Pain	Lizard meat is inserted in banana for easy swallowing and prescribed to eat	0.02	12.9
32	Reptile	Feti shap	Cobra	*Ophiophagus hannah* (Cantor, 1836) (V)	Elapidae	bile /gall bladder	Oral	Tonsil	Raw bile/gall bladder is prescribed to eat.	0.08	38.5
33	Mammal	Neola	Mongoose	*Herpestes edwardsii* (Saint-Hilaire, 1818) (V)	Herpestidae	Meat	Oral	Cancer	Meat is boiled and prescribed to eat	0.18	47.1
								Asthma			35.3
								Rabies			46.2
34	Mammal	Kerketuwa	Squirrel	*Sciurus caroliniensis* (Gmelin, 1788) (V)	Sciuridae	Meat	Oral	Asthma	Boiled meat is prescribed to eat	0.21	29.6
35	Mammal	Garu	Cow	*Bos indicus* (Linnaeus, 1758) (V)	Bovidae	Milk (curd)	Oral	Skin cancer (anduriya)	250 ml of curd with juice from the roots of Hun borial plant (*Sida cordifolia* L.) and sira of bakul bora rice prescribed to take for 3 days	0.40	66.7
						Milk	Oral	Chronic dysentery	Milk is mixed with juice of Sotiona plant (*Alstonia scholaris* L.) leaf and bark and prescribed to take for 3 days.		85.7
						urine	Oral	Epilepsy (Mrigi bemar)	Cow urine is mixed with crushed seed of Bokful (*Sesbania grandiflora* (L.) Pers.) and prescribed to drink		71.4
36	Mammal	Moh	Buffalo	*Bubalus bubalis* (Linnaeus, 1758) (V)	Bovidae	Horn	Oral	Pre menstrual pain	After burning of buffalo horn the ash is mixed with water and prescribed to drink	0.37	77.8
37	Mammal	Ketela pohu	Porcupine	*Hystrix indica* (Linnaeus, 1758) (V)	Hystricidae	Elementary canal	Oral	Pre menstrual pain	alimentary canal is dried and ground and then mixed with water and prescribed to drink	0.13	81.5
38	Mammal	Horin/Dolhorin	Deer/Swamp deer	*Rucervus duvaucelii or Cervus duvaucelii* (Cuvier, 1823) (V)	Carvidae	Horn	Topical	Piles	Horn of dear is burned and the smoke is used in piles region	0.08	63.2
39	Mammal	Sunga baduli	Indian fruit bat (vesper bat)	*Pipistrellus coromandrs* (Gray, 1838) (V)	Vespertilionidae	Blood	Oral	Vomiting	Blood is prescribed to drink to stop vomiting	0.05	61.5
40	Mammal	Bor Baduli	Bat	*Pteropus gigantus* (Brunnich, 1782), *Rhinolophus spp.* (Lacepede,1799) (V)	Pteropodidae	meat	Oral	Asthma	Meat is cooked and prescribed to eat	0.66	94.7
41	Mammal	Shial	Indian fox	*Vulpes bengalensis* (Shaw, 1800) (V)	Canidae	meat	Oral	Paralysis	Cooked meat is consumed	0.52	91.3
42	Mammal	Manuh	Human	*Homo sapiens* (Linnaeus, 1758) (V) *.*	Homonidae	urine	Oral	Senseless	Prescribed to drink human urine	034	75.0
							Topical	Wound	Urine is used as antiseptic in wound		47.8

*V* vertebrates, *IV* invertebrates, *RFC* relative frequency of citation, *FL* fidelity level

*The local names of the animals are given in Assamese Language

The use of a number of animals and animal derived drugs by different ethnic communities to treat different diseases have also been reported from different geographical regions in India. A total of 15 different animal species were reported to be used for therapeutic purposes by the Mogya, Bawaria, and Meena community of Rajasthan [54]. About 26 animal species were reported to be used by the Naga tribe of Nagaland [26] and 48 different animals were recorded and documented to be used for different ethnomedicinal purposes among the Karbis of Assam [31]. Different indigenous tribal groups also sacrifice animals for different rituals and religious purposes in keeping with their mythological myths and beliefs. For example, people wear bear and tiger claws around their neck to protect from evils while animals like goat, buffalo, pigeon were sacrificed to please Gods for healing purposes [54–56].

From the study conducted, treatment was found for assisting 40 different ailments such as asthma, pneumonia, cancer, fever, piles, gastric, diabetes, snake bite, Pox, otorrhoes etc. (Table 2). The use of whole animals for medicinal purpose was recorded to be the highest (44.9%), followed by other animals parts and byproducts like meat (22.5%), blood, head, alimentary canal, gall bladder/bile, urine, horn, milk (each 4.1%) and heart, cocoon with larva (each 2.0%).

**Fig. 3:**
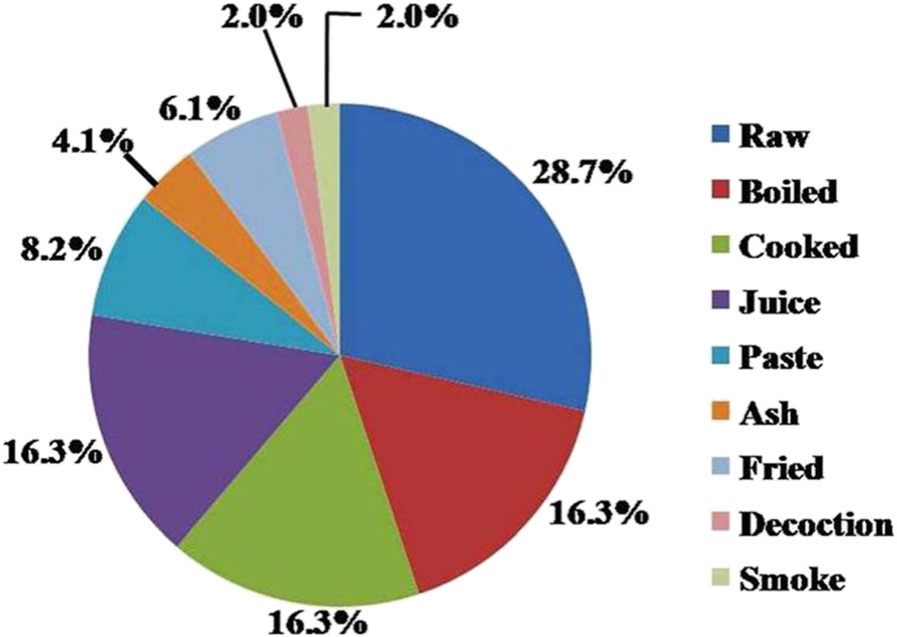
Methods of preparation of different animals and animals parts (%)

**Fig. 4:**
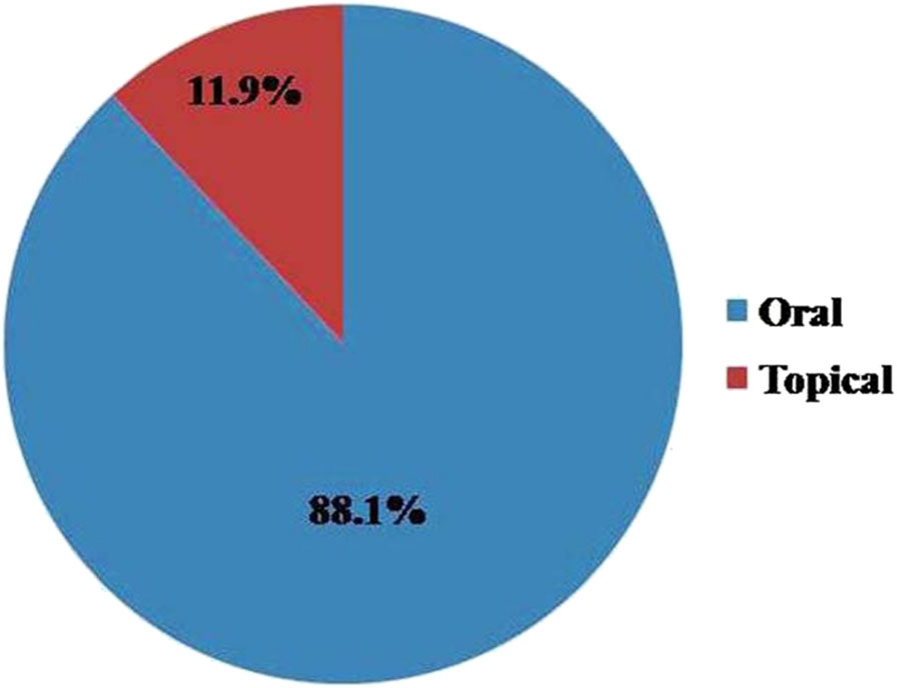
Percentage of mode of application

The result of our study depicted a total of 9 modes of preparation of animals for consumption against different ailments. The most commonly used way of taking animals in the study area is by consuming raw which occupied 28.7% of total preparation, followed by boiled, cooked and juice (each 16.3%), paste (8.2%), Ash (4.1%), fried (6.1%), decoction and smoke (each 2.0%) (Fig. 3). Raw consumption of animals or animal parts in different therapeutic purposes is a common practice among different ethnic communities worldwide [2, 57]. Further, it was noted that the oral consumption of the preparations to treat the ailments were much higher (88.1%) than that applied topically (11.9%) (Fig. 4). This is quite similar to the observation made in other studies [31, 57–59]. However, topical application is still a most important way of remedy to treat disease like pain, bone fracture, wound, piles, otorrhoea etc. [31, 57, 58].

**Fig. 5:**
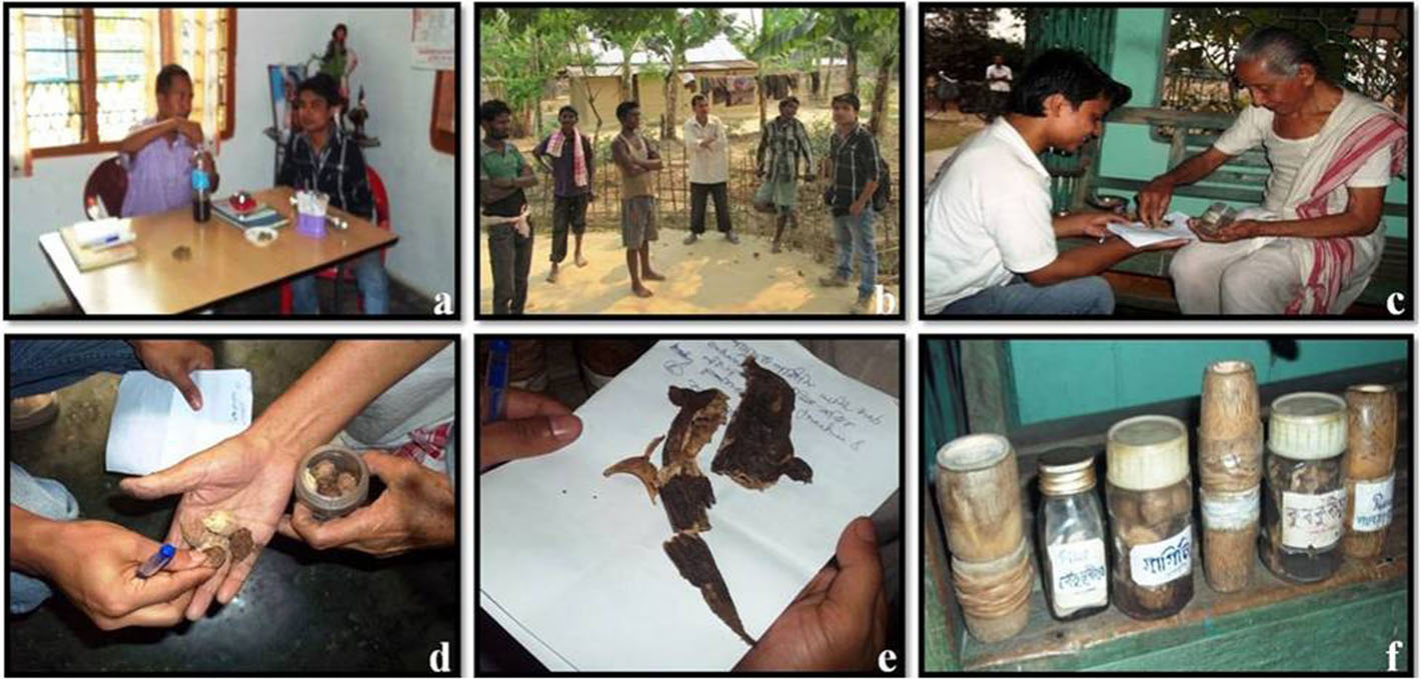
Representative photographs during field survey showing traditional method of drug preparation by the local traditional healers with one of the authors (MPB). **a**, **b**, **c** Interaction and data collection from traditional healers. **d** Dry cocoon of *Mantis religiosa*; **e** Dry fish *Chaca chaca*; **f** Traditional stockes and jars of animals and animal parts

The study also showed that for better therapeutic and remedial purposes plants and plant derived products were also used in combination with the animal parts and byproducts to treat some ailments (Table 2). For example, cow urine is mixed with crushed seed of *Sesbania grandiflora* (L.) Pers. (Bokful) for the treatment of epilepsy, cow milk is mixed with juice of *Alstonia scholaris* L. (Sotiona) leaves and bark to treat chronic dysentery etc. Some medicinal preparation where both plant and animals are utilized in combination is also reported from Brazil [7, 9, 60]. Some of the animal species being used by these ethnic groups, have also been reported to be used for similar purpose elsewhere. Cockroach (*Periplaneta americana*), found to treat asthma in our study site, has also been reported to have the same usage in Brazil [61]. Similarly Mishra and Panda [62] have documented the use of cockroach excreta in the treatment of bronchitis from coastal region of Orissa, India. Moreover, the present findings recorded the use of *Hystrix indica* elementary canal in the treatment of pre-menstrual pain, where as elementary canal of *Hysrix indica* was reported to be used as antiasthmatic in cough and cold among Pahari and Danawar tribe of central Nepal [63]. It was also noted that honey bees were used against treatment of cancer suspects and this observation could be in line with the findings of Jo et al. [64] where honey bee venom toxin and melittin were suggested for anticancer effect in ovarian cancer cells through induction of death receptor and inhibition of JAK2/STAT 3 pathway. Human urine was documented in the present study to be used against wound healing and recovery from senseless, however, Verma et al. [31] have reported the use of human urine as a therapeutic against conjunctivitis and skin diseases by Karbi community of Assam, India. According to Zhang et al. [65], the medicinal usage of earthworms in China has a history of nearly 4000 years for the treatment of 80 different diseases like asthma, epilepsy, cancer etc. Present study also documents the use of earthworms against many diseases like vocal cord infection, piles, cancer, and pneumonia. Pharmacological importance of earthworms is also supported by the study of Dinesh et al. [66], where they showed the anticancer potential of peptides of coelomic fluid of earthworms. However, special precaution should be taken when animal tissue or parts from unknown sources are used as remedies due to possibility of transmission of severe and prevalent zoonoses and other side effects. Photographs captured during field survey showing the interaction with some traditional healers and some representative animals, animal parts and their storing method is given in Fig. 5.

### Quantitative analysis

#### Relative frequency of citation (RFC)

Relative frequency of citation (RFC) index was calculated to determine the local importance of each species. The most cited animal species were: *Metaphire houletti/Pheretima posthum* (RFC = 0.68), *Pteropus gigantus/Rhinolophus sp*. (RFC = 0.66), *Amphipnous cuchia* (RFC = 0.66), *Periplaneta Americana* (RFC = 0.65), *Clarius batrachus* (RFC = 0.59), *Pila spp*. (0.58), *Vulpes bengalensis* (0.52). The highest value of RFC index was scored by *Metaphire houletti/Pheretima posthum* which demonstrates the importance of this animal species in adjoining areas of Gibbon wildlife sanctuary, Assam, India as it was mentioned by a higher number of informants. However, animal species with low RFC values for instance *Hemidactylus flaviviridis* (RFC = 0.02) and *Pherosphus sp.* (RFC = 0.02) do not mean that they are not important locally but it may be that the most of the people are not aware of their therapeutic properties.

#### Fidelity level (FL)

Fidelity level is very helpful for identifying most frequently and preferably used species in the treatment of certain disease. This fidelity level varies from 1.0% to 100% on the basis of respondents claiming the use of certain animals for the same purpose. A higher FL of 100% or close to 100% for a specific animal indicates that all of the used reports mentioned the same method for using the animal for treatment for the same diseases [57]. The present study showed 5 animal species with a FL above 90% (Table 2, Sl. No. 3,10,15, 24,41) such as *Perplaneta americana* which are used for the treatment of asthma (FL ~ 92.3%), *Perionyx sp*. used for treatment of pneumonia (F L ~ 95.2%), *Amphipnous cuchia* for treatment of anemia (FL ~ 90.9%), *Vulpes bengalensis* used for treatment of paralysis (FL ~ 91.3%) and *Antheraea assamensis* used as food for highly proteinacious contents with a FL 100%. However *Pherosophus sp.* has the lowest fidelity level (FL ~ 6.9%). Observably, the remedies for frequently reported ailments have the maximum fidelity level and those with less number of reports have lowest FL values. From this study, the results indicate that in many cases same animal species were reported to be used for the healing of more than one ailment. This type of trends has also been found in different traditional medicinal remedies in different parts of the world [14, 23, 44, 67]. On the other hand, different animal species were sometimes used to treat the same disease. The use of different animals or remedies for the same ailment is popularly valued as it provides an adaptation to the availability and accessibility of the possible animals [68].

In summary, this study indicates that traditional zootherapeutic practices play an important role in the primary health care system among the indigenous ethnic communities inhabiting near the Gibbon Wildlife Sanctuary, Assam, India. Due to lack of proper medical facilities and due to the strong belief on traditional medicine these people depend on various animal based medicines for different therapeutic purposes. It is necessary to take care of ecological balance and biodiversity conservation measure in terms of uses and sale of animals and animal byproducts for medicinal purpose. Many superstitions and myth may also be associated with traditional medicine, therefore, particular animal or its part, byproduct should be tested for their appropriate medicinal component. Further, due to death of elderly knowledgeable persons and rapid modernization, the traditional zootherapeutic knowledge is eroding and findings from present study should be helpful to preserve and document the knowledge of these ethnic groups on zootherapeutic usages for future.

## Conclusion

Use of animals and animal derived products for indigenous medicinal purposes in the study site is the main primary health care system. This study is the first effort to document the traditional zootherapeutic knowledge common among the indigenous inhabitants surrounding Gibbon Wildlife Sanctuary. Traditional knowledge is not only significant for its pharmacological value, but also related with different cultural beliefs and sentiments of the indigenous people. This study provides the base for further scientific validation of the therapeutic efficacy of various zootherapeutic tradiotional uses by these people and finding novel biological compound(s) towards discovery of new drugs. This may also help in better understanding of traditional zootherapeutic medicine, its interrelationship with the socioeconomic and ecological values of the region, biodiversity conservation and management strategies of animal resources for sustainable use.
